# Rapid Synthesis of Noble Metal Colloids by Plasma–Liquid Interactions

**DOI:** 10.3390/ma17050987

**Published:** 2024-02-21

**Authors:** Yuanwen Pang, Hong Li, Yue Hua, Xiuling Zhang, Lanbo Di

**Affiliations:** 1 College of Physical Science and Technology, Dalian University, Dalian 116622, China; pangyuanwen2021@163.com (Y.P.); huayuexx@163.com (Y.H.); xiulz@sina.com (X.Z.); 2 State Key Laboratory of Structural Analysis for Industrial Equipment, Dalian University of Technology, Dalian 116024, China; 3 Key Laboratory of Advanced Technology for Aerospace Vehicles of Liaoning Province, Dalian University of Technology, Dalian 116024, China

**Keywords:** noble metal colloids, surface DBD plasma, plasma–liquid interactions, hydrogen peroxide

## Abstract

The interactions between plasma and liquids cause complex physical and chemical reactions at the gas–liquid contact surface, producing numerous chemically active particles that can rapidly reduce noble metal ions. This study uses atmospheric-pressure surface dielectric barrier discharge (DBD) plasma to treat ethanol aqueous solutions containing noble metal precursors, and stable gold, platinum, and palladium colloids are obtained within a few minutes. To evaluate the mechanism of the reduction of noble metal precursors by atmospheric-pressure surface DBD plasma, the corresponding metal colloids are prepared first by activating an ethanol aqueous solution with plasma and then adding noble metal precursors. It is found that the long-lived active species hydrogen peroxide (H_2_O_2_) plays a dominant role in the synthesis process, which has distinct effects on different metal ions. When HAuCl_4_ and H_2_PdCl_4_ are used as precursors, H_2_O_2_ acts as a reducing agent, and AuCl_4_^−^ and PdCl_4_^2−^ ions can be reduced to metallic Au and Pd. However, when AgNO_3_ is the precursor, H_2_O_2_ acts as an oxidising agent, and Ag^+^ ions cannot be reduced to obtain metal colloids because metallic Ag can be dissolved in H_2_O_2_ under acidic conditions. A similar phenomenon was also observed for the preparation of Pd colloid-PA with a plasma-activated ethanol aqueous solution using Pd(NO_3_)_2_ as a Pd precursor.

## 1. Introduction

Noble metal nanoparticles have shown great potential for applications in various fields such as catalysis [1,2,3], sensing [4], fuel cells [5], and biomedicine [6,7] because of their high specific surface area, unique optoelectronic properties, and ease of synthesis [8]. Controlling the synthesis of nanoparticles, particularly colloidal dispersions for the preparation of noble metal nanoparticles, is essential for developing new custom catalysts for energy conversion and industrial and medical applications [9].

Noble metal colloids are usually prepared by chemical, physical, biosynthetic, and plasma methods. Traditional chemical methods use chemical reducing agents, such as sodium citrate [10], NaBH_4_ [11], and ascorbic acid [12] mixed with noble metal salt precursors to synthesise noble metal colloids. The size and morphology of nanoparticles can be altered by changing the amount of organic solvent and reagent used in the preparation process. However, these chemical reduction agents are highly toxic and easily pollute the environment, which is not conducive to sustainable development and limits scale expansion [13]. Physical methods, such as laser ablative synthesis in a solution or ion beam sputtering, can also be used to prepare metal colloids; however, their equipment is expensive and energy-intensive [14]. The process of biosynthesis involves biological systems such as bacteria, fungi, and plant extracts, which are environmentally friendly and economical but also have drawbacks, including slow synthesis rates, limited material compatibility, pollution and purity, and variability between batches [15]. Compared with these traditional noble metal nanoparticle synthesis methods, the plasma synthesis process is simple and has high reactivity, which can quickly generate noble metal nanoparticles [16,17,18] and promote the phase transition and redispersion of metal nanoparticles in the later stage [19,20].

Many studies are dedicated to utilising plasma for the preparation of metal nanoparticles. One commonly used method is direct current glow discharge. For example, Dzimitrowicz et al. successfully synthesised fructose-stabilised, uniform, and monodisperse AgNPs through direct current glow discharge [21]. Li et al. successfully prepared nanoparticles using direct current glow discharge plasma, which was also able to rapidly and efficiently synthesise carbon quantum dots [22]. Although these methods have achieved success on a laboratory scale, scaling up for large-scale production still poses challenges. Another solution plasma produced by two electrodes immersed in liquid can also prepare nanoparticles, but it is also not suitable for amplification and will consume the electrode and even cause contamination of the final product of the electrode material [23]. Surface dielectric barrier discharge (DBD) is a typical non-equilibrium plasma at atmospheric pressure that can generate discharge at normal pressure and near room temperature without a vacuum. The high-energy electrons generated during discharge collide with the surrounding gas molecules, which can excite, dissociate, and ionise the gas molecules, producing numerous reactive radicals, excited atoms, molecules, and ions required for chemical reactions [24,25,26]. They are widely used in the preparation of highly dispersed metal nanoparticles because of the strong reducing power of the high-energy electrons (usually in inert gas plasmas such as Ar and He) or the hydrogen species in hydrogen-containing plasmas such as Ar/H_2_ discharges [27,28]. The former reduces metal ions with positive standard redox potentials such as Au, Ag, Pt, Pd, Ir, and Rh, whereas the latter is more efficient than electron reduction. It not only reduces metal ions with positive standard redox potentials but also some metal ions with negative standard redox potentials, such as Co and Ni [29].

The synthesis of noble metal nanoparticles usually uses colloidal methods [30]. Therefore, the interaction between the plasma and liquid is significant, and there are many complex physical and chemical processes at the interface of the plasma and liquid contact [31]. When the plasma–liquid system contains an ethanol aqueous solution, ethanol and water molecules participate in the gas discharge plasma through different processes, namely, sputtering, electric-field-induced hydrated cation emission, and evaporation [32]. Isomeric radicals of C_2_H_5_O (CH_3_CHOH, CH_2_CH_2_OH, and CH_3_CH_2_O), atomic hydrogen (H), and hydroxyl (OH) radicals can be generated by electron impact in the plasma [33]. Subsequently, the generated species are transported from the gaseous plasma to the liquid. In the positive half cycle of the discharge, the liquid acts as a cathode, and positive ions are accelerated across the cathode sheath to bombard the liquid surface, followed by evaporation, sputtering, and secondary electron emission from the liquid. In the negative half cycle, electrons are pulled out of the plasma to enter the liquid by the small electric field near the liquid anode. When these electrons enter the aqueous solution, they are hydrated to form the e^−^_aq_ species (with a standard redox potential of −2.8 V) in the solution. In addition, photons emitted by excited particles in plasma may enter the liquid, triggering secondary processes, and neutral particles in the plasma are transported to the liquid surface by diffusion. Therefore, the synthesis process can be controlled in both the solution and plasma phases by controlling the solution and plasma parameters [34,35].

Generally, active species generated in plasma and solution systems can be divided into short- and long-lived species based on their lifetimes [36]. Short-lived active species include free electrons, hydrated electrons (e^−^_aq_), excited hydrogen atoms (H), hydroxide radicals (OH), negative hydrogen ions (H^−^), and alcohol fragment radicals induced to dissociate from ethanol molecules by high-energy electrons or ultraviolet radiation, which disappear or rapidly decay after the plasma stops [37]. The long-lived active species is mainly H_2_O_2_, which can remain in liquids for a long time and can be detected within a few months of plasma irradiation [38]. Bjelajac et al. [39] synthesised AuNPs in a chloroauric acid solution with ethanol as a solvent using a DBD plasma torch at atmospheric pressure, and the results showed that plasma was conducive to the synthesis of more dispersed Au nanoparticles. Sauvageau et al. [40] synthesised platinum-group metal nanoparticles using a DBD hydrogen plasma and found that all three ions (Pt, Pd, and Rh) had high reduction rates.

In this study, atmospheric-pressure surface DBD cold plasma was developed and adopted to synthesise noble metal colloids by treating various metal precursor solutions. The results show that colloids of gold, platinum, and palladium can be successfully prepared by plasma in a few minutes. The synthesis process is fast, without the use of any other chemical reducing agent, and the prepared noble metal colloids have no significant change after 30 days of storage at room temperature. Moreover, in order to investigate the reduction mechanism of noble metal precursors by surface DBD plasma at atmospheric pressure, we synthesised noble metal colloids using both direct plasma treatment and plasma-activated ethanol aqueous solution treatment of the noble metal precursors, and a comparative analysis was then conducted. It was found that the difference in the metal colloids prepared by the two methods can be attributed to the distinct active species present in the solution, and the effect of these active species varies depending on the specific metal precursor employed.

## 2. Experimental Section

### 2.1. Materials

Gold trichloride (AuCl_3_·HCl·4H_2_O), chloroplatinic acid (H_2_PtCl_6_·6H_2_O), palladium chloride (PdCl_2_), palladium nitrate (Pd(NO_3_)_2_·2H_2_O), silver nitrate (AgNO_3_), and anhydrous ethanol (AR, ≥99%) used in this experiment were purchased from Kermel Chemical Reagent Co, Ltd. (Tianjin, China). Gold trichloride was dissolved in water before use to obtain an aqueous solution of chloroauric acid (HAuCl_4_) with a concentration of 20 mM, and palladium chloride was dissolved in hydrochloric acid before use to obtain a solution of chloropalladic acid (H_2_PdCl_4_) with a concentration of 521 mM. Polyvinylpyrrolidone (PVP) (Mw 58000, K29-32) was purchased from Aladdin Chemical Reagent Co, Ltd. (Shanghai, China). High-purity argon (>99.999%) and hydrogen (>99.999%) were purchased from Zhonghao Guangming Chemical Research and Design Institute Co, Ltd. (Dalian, China).

### 2.2. Preparation of Noble Metal Colloids

#### 2.2.1. Surface Dielectric Barrier Discharge (DBD) Reactor

**Figure 1:**
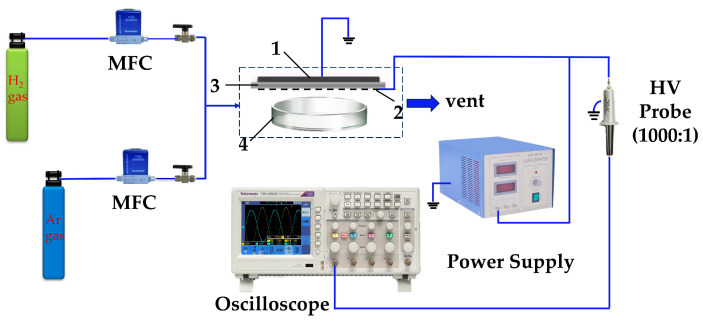
Diagram of the experimental setup for the preparation of noble metal colloids by surface dielectric barrier discharge: 1—grounding electrode, 2—high-voltage electrode, 3—dielectric layer, 4—noble metal precursor solution.

The surface DBD reactor consists of a high-voltage electrode and a grounding electrode separated by a high-purity alumina dielectric layer (area: 9 × 5 cm^2^; thickness: 1 mm), and the experimental setup is shown in Figure 1. Both electrodes are made of high-purity tungsten, the size of the grounding electrode is 1.7 × 0.5 cm^2^, and the high-voltage electrode consists of nine comb-like tungsten wires connected at one end (tungsten width of 1 mm, wire spacing of 4 mm). The discharge voltage was measured by a high-voltage probe (Tektronix P6015A, Beaverton, OR, USA).

#### 2.2.2. Direct Preparation of Au Colloid-P, Pt Colloid-P, and Pd Colloid-P with Plasma

First, 2 mL of an ethanol aqueous solution (50% water, 50% ethanol) containing 5% PVP was prepared, and then 25 μL of HAuCl_4_ (20 mM) was added to it, and the precursor mixture solution was mixed thoroughly. The mixed solution was placed in a quartz reactor (3 cm diameter and 4 mm depth), and the height of the quartz reactor was adjusted such that the liquid level was 2 mm below the high-voltage electrode. The working gas was a mixture of Ar and H_2_ with a total flow rate of 100 sccm and an Ar/H_2_ molar ratio of 1:1. The rotational speed of the magnetic stirrer was set to 500 r·min^−1^, the peak-to-peak sinusoidal applied voltage was 9.0 kV, the discharge frequency was 10.4 kHz, and the plasma treatment was carried out for 7 min. A wine-red solution was obtained after the plasma treatment. The solution was poured into a 5 mL measuring cylinder, and ethanol was added to compensate for the loss of liquid during the plasma treatment until the total volume of the mixed solution was 2 mL. After mixing, the solution was poured into a sample bottle, labelled as Au colloid-P, and stored in a refrigerator at 4 °C in the dark. The preparation processes for Pt colloid-P and Pd colloid-P were the same. H_2_PtCl_6_ and H_2_PdCl_4_ were used as noble metal precursors in the preparation process.

#### 2.2.3. Preparation of Au Colloid-PA and Pd Colloid-PA with Plasma-Activated Solution

First, an ethanol aqueous solution containing 5% PVP (50% water, 50% ethanol, 2 mL) was prepared in a quartz reactor (diameter: 3 cm; depth: 4 mm), underwent plasma treatment (treatment conditions were consistent with Section 2.2.2), and then 25 μL of HAuCl_4_ (20 mM) was added without plasma. The colour of the mixture quickly turned wine-red, indicating the presence of AuNPs. The obtained mixed solution was denoted as Au colloid-PA and stored in a refrigerator at 4 °C in the dark. The preparation process for Pd colloid-PA was the same. H_2_PdCl_4_ and Pd(NO_3_)_2_ were chosen as palladium precursors in the preparation process.

### 2.3. Characterisation

A UV-Vis spectrometer (Hitachi, U-3900, Tokyo, Japan) was used to record the absorption of the sample in the wavelength range of 200–800 nm. Sample analysis was performed using transmission electron microscopy (TEM) and high-resolution transmission electron microscopy (HRTEM) (JEOL JEM-2100F, Tokyo, Japan) at an acceleration voltage of 120 kV. More than 100 Au NPs, Pt NPs, and Pd NPs were selected from the corresponding TEM images of noble metal colloids, and the particle size and distribution of the three noble metal colloids were calculated.

## 3. Results and Discussion

### 3.1. Light-Absorbing Characteristics and Stability of Metal Colloids Prepared with Plasma

**Figure 2:**
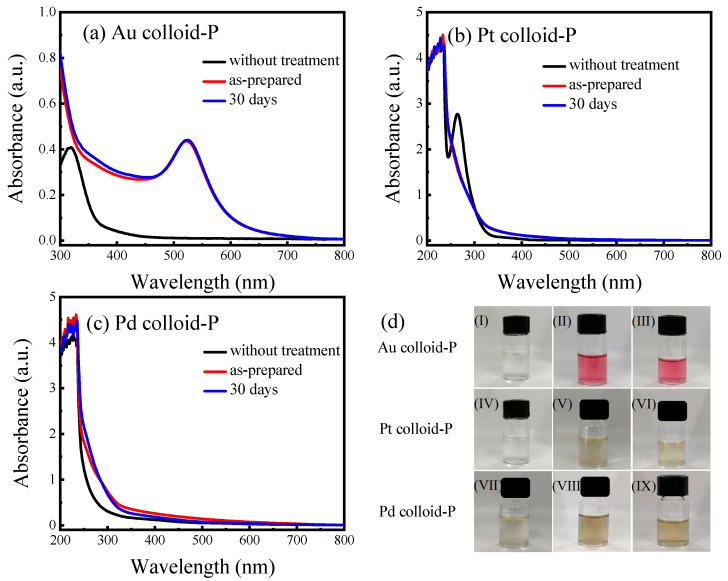
UV-Vis absorption spectra of (**a**) Au colloid-P, (**b**) Pt colloid-P, and (**c**) Pd colloid-P (using H_2_PdCl_4_ as Pd precursor) prepared by surface DBD cold plasma at atmospheric pressure and after 30 days of storage, and (**d**) corresponding photos before and after treatment and after 30 days of storage (gold colloids: (I)–(III); platinum colloids: (IV)–(VI); palladium colloids: (VII)–(IX)).

Using atmospheric-pressure surface DBD plasma at a discharge voltage of 9 kV and a discharge time of 7 min, solutions of chloroauric acid, chloroplatinic acid, and chloropalladic acid were treated separately to prepare gold colloids (Au colloid-P), platinum colloids (Pt colloid-P), and palladium colloids (Pd colloid-P), which were the most stable (Figures S1–S4). The UV–Vis absorption spectra and corresponding photographs of the three untreated metal precursor solutions and three freshly prepared metal colloids are shown in Figure 2. Figure 2a shows that the untreated chloroauric acid solution (0.25 mM) exhibited only weak absorption in the visible light region, and the sample colour appeared light yellow ((I) in Figure 2d). In comparison, the fresh Au colloid-P samples prepared by the plasma treatment showed obvious surface plasmon resonance (SPR) absorption peaks of gold at 500–550 nm, and the colour of the samples was burgundy ((II) in Figure 2d). Meanwhile, the maximum SPR absorption peak of the Au colloid-P sample appeared at 521 nm, indicating that the particle size of the AuNPs was well controlled below 20 nm. As shown in Figure 2b, the untreated chloroplatinic acid solution (0.25 mM) exhibits significant absorption in the UV wavelength range of 200–300 nm, corresponding to the characteristic absorption band of a PtCl_6_^2−^ ion. The sample showed very weak absorption in the visible light region and appeared light orange ((IV) in Figure 2d). After plasma treatment, the Pt colloid-P sample was obtained, and the characteristic absorption band of an PtCl_6_^2−^ ion disappeared. The sample showed weak absorption in the visible light region, and the colour of the sample turned brown ((V) in Figure 2d), indicating that the plasma treatment can reduce PtCl_6_^2−^ ions to a Pt elemental substance. As shown in Figure 2c, the untreated chloropalladic acid solution (0.25 mM) exhibited weak absorption in the visible light region, and the sample appeared light brown ((VII) in Figure 2d). After plasma treatment, the Pd colloid-P sample was obtained, and its absorption intensity in the ultraviolet and visible light regions was slightly enhanced compared to the untreated H_2_PdCl_4_ solution, and the sample colour turned brown ((VIII) in Figure 2d), indicating that plasma treatment can reduce chloropalladic acid to Pd.

To study the stability of the plasma-prepared Au colloid-P, Pt colloid-P, and Pd colloid-P samples, the three samples were stored for 30 days and re-characterised by UV-Vis absorption spectroscopy, and the corresponding photographs were taken, as shown in Figure 1. Compared to the freshly prepared samples, after 30 d of storage, there was no significant change in the position and intensity of the absorption peaks in the UV–Vis absorption spectra of the three samples, and no precipitation was observed, indicating that the plasma-prepared Au colloid-P, Pt colloid-P, and Pd colloid-P samples had good stability.

### 3.2. Morphology and Particle Size of Metal Colloids Prepared with Plasma

**Figure 3:**
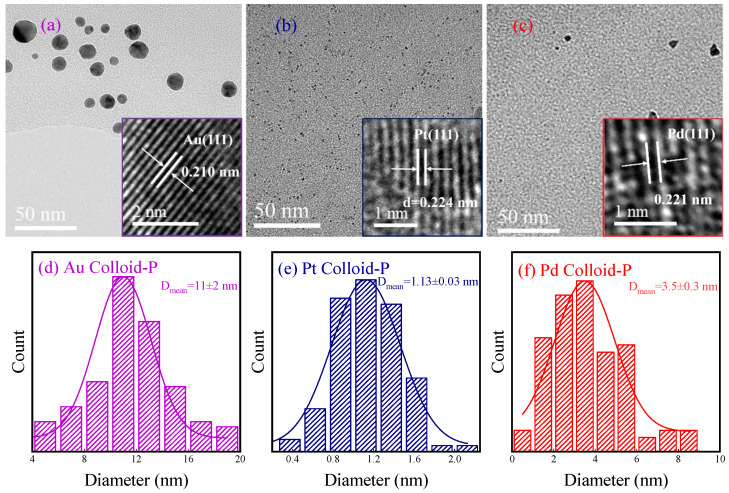
TEM photographs and HRTEM photographs (insets) of (**a**) Au colloid-P, (**b**) Pt colloid-P, and (**c**) Pd colloid-P and (**d**–**f**) histograms of the particle size distribution of the corresponding metal nanoparticles.

Figure 3a–c show the transmission electron microscopy (TEM) and high-resolution transmission electron microscopy (HRTEM) images of the Au, Pt, and Pd colloid-P samples. From the figures, it can be observed that the metal nanoparticles in the three samples have good dispersibility and are in a spherical state, without obvious agglomeration. This is because PVP has a good stabilising effect on the metal nanoparticles in the system. Figure S5 shows that the prepared gold colloids are unstable when no PVP is present in the system. In addition, the HRTEM images of the three samples show clear lattice stripes, indicating that the metal nanoparticles in the prepared samples have a good degree of crystallisation. According to the HRTEM images, the lattice spacings of the Au colloid-P, Pt colloid-P, and Pd colloid-P samples were 0.210, 0.224, and 0.221 nm, corresponding to the Au (111), Pt (111), and Pd (111) crystal planes, respectively, all of which belong to the face-centred cubic structure (Fcc). Based on the TEM photographs of the samples, histograms of the particle size distribution of the metal nanoparticles of the Au colloid-P, Pt colloid-P, and Pd colloid-P samples were obtained by selecting more than 100 Au, Pt, and Pd nanoparticles, respectively, as shown in Figure 3d–f. In the Au colloid-P, Pt colloid-P, and Pd colloid-P samples, the average particle sizes of Au, Pt, and Pd are 11.0 ± 2.0 nm, 1.1 ± 0.1 nm, and 3.5 ± 0.3 nm, respectively. The larger size of the Au nanoparticles was mainly due to the influence of chloride ions in the precursor HAuCl_4_ solution [41]. However, the particle sizes of Pt and Pd are very small, especially Pt, with a particle size of only 1.1 ± 0.1 nm, which is very suitable for use as metal catalysts [42,43].

### 3.3. Light Absorption Properties and Morphology of Au Colloid-PA Prepared with Plasma-Activated Solution

**Figure 4:**
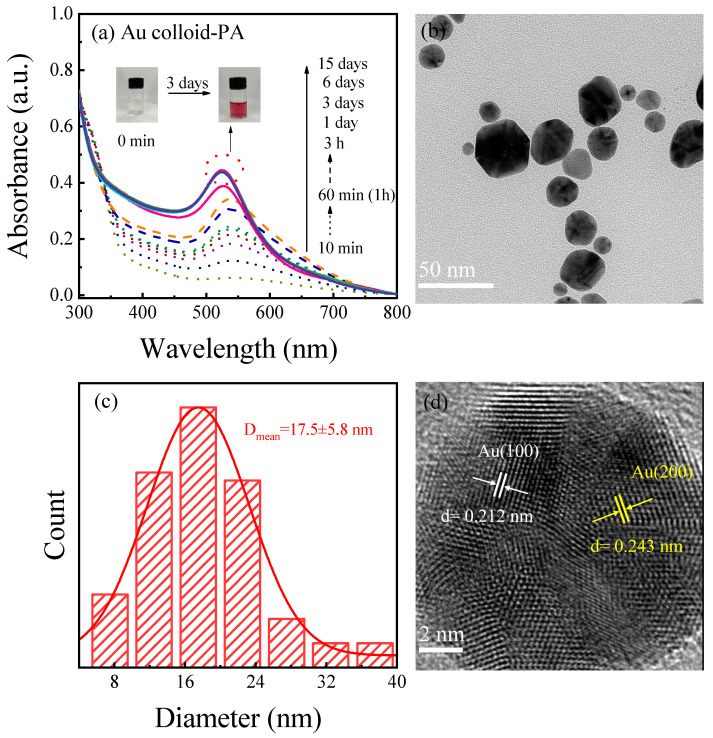
(**a**) UV-Vis absorption spectra of Au colloid-PA solution left from 10 min to 15 d, (**b**) TEM image of Au colloid-PA and corresponding (**c**) histogram of Au nanoparticle size distribution, (**d**) HRTEM image.

To evaluate the light-absorbing characteristics and stability of the Au colloid-PA samples prepared from the plasma-activated solutions, UV-Vis absorption spectra were used to record the changes in their absorption spectra over a period of 15 d, as shown in Figure 4a. The figure shows that the SPR absorption peak of gold nanoparticles appeared at approximately 532 nm after adding HAuCl_4_ to the plasma activation solution for only 10 min. With time, the position of the SPR absorption peak blue-shifted, and the intensity of the absorption peak gradually increased. Three days later, the SPR absorption peak of the Au colloid-PA sample blue-shifted to 528 nm, the intensity of the absorption peak no longer changed, and the sample appeared purplish-red (Figure 4a). Meanwhile, within 3–15 days, there was no significant change in the absorption peak intensity, position, or colour of the Au colloid-PA sample, indicating that the plasma activation solution could also reduce chloroauric acid to prepare metallic gold nanoparticles, and the stability of the prepared Au colloidal solution was good.

To compare the morphology and size of the gold nanoparticles in the plasma-activated solution-prepared Au colloid-PA and plasma-directly prepared Au colloid-P samples, the Au colloid-PA sample was characterised using TEM and HRTEM, and the results are shown in Figure 4b,d. The gold nanoparticles in the Au colloid-PA sample were well dispersed and did not undergo significant aggregation. This is because PVP plays a stabilising role in the colloidal solution of the system. Unlike the spherical gold nanoparticles in the Au colloid-P sample, the Au nanoparticles in the Au colloid-PA sample exhibited spherical, triangular, and hexagonal shapes. From Figure 4d, it can be observed that the lattice fringes in the HRTEM image of the Au colloid-PA sample are clear, with good crystallinity and two types of crystal planes: (111) and (200). By selecting more than 100 gold nanoparticles for the analysis, the average particle size of the gold nanoparticles in the Au colloid-PA sample was found to be 17.5 ± 5.8 nm (Figure 4c), which is approximately 6.5 nm larger than the average particle size of Au nanoparticles directly prepared by plasma in the Au colloid-P sample. This may be due to the electrostatic repulsion generated under direct plasma treatment, which hinders the aggregation and growth of AuNPs, whereas the long-lived active species in the plasma-activated solution slowly reduce HAuCl_4_, resulting in weaker electrostatic repulsion and the generation of gold nanoparticles with different morphologies and larger particle sizes.

### 3.4. Mechanism of Plasma Preparation of Metal Colloids

**Figure 5:**
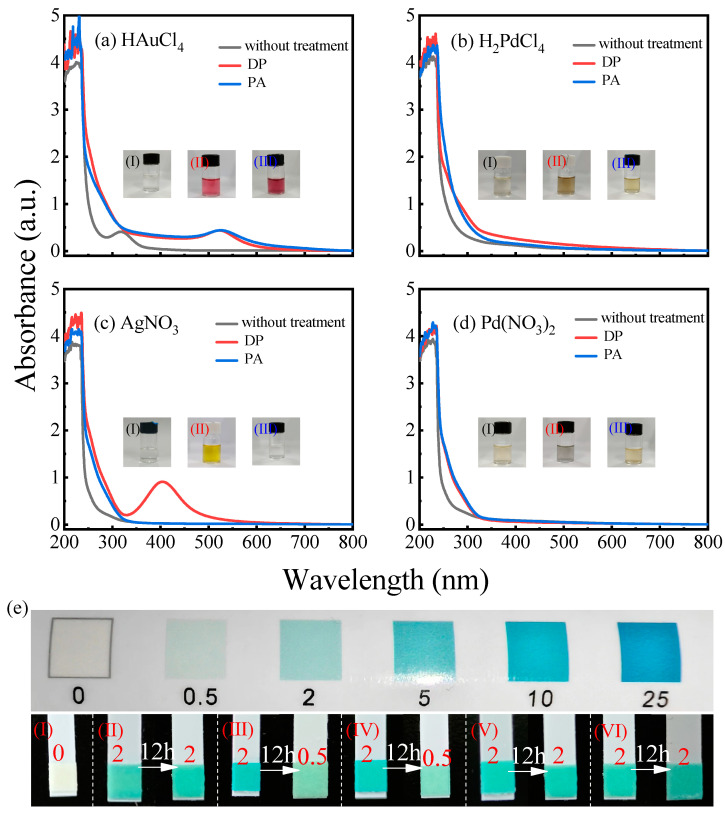
UV-Vis absorption spectra of (**a**) HAuCl_4_, (**b**) H_2_PdCl_4_, (**c**) AgNO_3_, and (**d**) Pd(NO_3_)_2_ under different treatments; (**e**) results of hydrogen peroxide detection strips: (I) untreated ethanol aqueous solution and (II) ethanol aqueous solution treated with plasma activation and left for 12 h. Results of adding (III) HAuCl_4_, (IV) H_2_PdCl_4_, (V) AgNO_3_, and (VI) Pd(NO_3_)_2_ to ethanol aqueous solution treated with plasma activation and left for 12 h. (H_2_O_2_ concentration unit: mg/L).

Figure 5a–d show the UV–Vis absorption spectra and corresponding photographs of the untreated HAuCl_4_, H_2_PdCl_4_, AgNO_3_, and Pd(NO_3_)_2_ precursor solutions and the corresponding metal colloids prepared by direct plasma (DP) treatment and plasma activation (PA) treatment. It can be seen that the HAuCl_4_, H_2_PdCl_4_, AgNO_3_, and Pd(NO_3_)_2_ precursors can all be reduced to obtain the corresponding noble metal colloid by DP treatment. This is because during the DP treatment process, abundant short-lived reducing species can be continuously generated, such as free electrons, hydrated electrons (e^−^_aq_), excited hydrogen atoms (H), and reactive alcohol fragment radicals generated by high-energy electrons or the UV-induced dissociation of ethanol molecules, as well as the long-lived active species hydrogen peroxide. These reactive radicals have a lower redox potential and can effectively reduce the precursor solution ions to elemental nanoparticles.

Nevertheless, only HAuCl_4_ and H_2_PdCl_4_ precursors can be reduced to obtain gold and palladium colloids by PA treatment. Meanwhile, AgNO_3_ and Pd(NO_3_)_2_ precursors cannot be reduced to obtain the corresponding metal colloids. This can be attributed to the effect of the residual long-lived species of H_2_O_2_, while the short-lived reducing species disappeared in the plasma-activated solution. HAuCl_4_ can be reduced by the long-lived active species H_2_O_2_ via the following reaction [44]:

(1) $$2{{\mathrm{AuCl}}_{4}}^{-}+3H_{2}O_{2}\;\to \;2\mathrm{Au}+3O_{2}+6H^{+}+8{\mathrm{Cl}}^{-}$$

The reduction mechanism of H_2_PdCl_4_ is similar to that of HAuCl_4_. And the long-lived species of hydrogen peroxide in solution may be produced by OH radicals generated in the gas-phase plasma dissolved in the liquid, as shown in Equations (2)–(6) [33,45].

(2) $$H_{2}O_{g}\overset{plasma}{\to }{OH}_{g}+H_{g}$$

(3) $$C_{2}{H_{5}OH}_{g}\overset{plasma}{\to }{C_{2}H}_{5g}+{OH}_{g}$$

(4) $${CH}_{3}CHOH\overset{plasma}{\to }{C_{2}H}_{4g}+{OH}_{g}$$

(5) $${OH}_{g}⟶{OH}_{aq}$$

(6) $${OH}_{aq}+{OH}_{aq}⟶H_{2}O_{2aq}$$

Studies have shown that both Ag^+^ reduction and oxidation processes occur during AgNP synthesis [46,47]. The e^−^_aq_, H, and alcohol fragment radicals are the main reducing agents for Ag^+^ to AgNPs, whereas H_2_O_2_ generated by the plasma–liquid interaction oxidises AgNPs to Ag^+^, as shown in Equation (7).

(7) $${\mathrm{Ag}}^{+}\overset{e_{aq}^{-}/H_{2}O_{2}}{↔}\mathrm{Ag}$$

Because e^−^_aq_, H, and reactive alcohol fragment radicals are short-lived species that rapidly decay or disappear after plasma quenching, the long-lived species H_2_O_2_, in an acidic environment, exerts etching effects on Ag, preventing the formation of AgNPs even in the presence of ethanol in the solution [48]. Hence, AgNO_3_ treated with plasma activation (PA) cannot obtain silver colloids. The inability to obtain palladium colloids by adding Pd(NO_3_)_2_ to plasma-activated ethanol aqueous solution may be attributed to the strong oxidising effect on PdNPs by H_2_O_2_ under acidic conditions combined with the existence of NO_3_^−^.

To verify the presence of H_2_O_2_ in the plasma activation solution and whether H_2_O_2_ species react during the synthesis process, we used H_2_O_2_ detection strips to test the solutions before and after plasma activation; the results are shown in Figure 5e. The untreated ethanol aqueous solution could not change the colour of the H_2_O_2_ detection strips ((I) of Figure 5e), whereas the plasma-activated ethanol aqueous solution turned the H_2_O_2_ detection strips into light green, and there were no significant changes after 12 h ((II) of Figure 5e), indicating that the long-lived species H_2_O_2_ was formed in the solution during the plasma treatment. When HAuCl_4_ and H_2_PdCl_4_ were added to the activated solution, the colour of the hydrogen peroxide detection strips slightly deepened ((III) and (IV) of Figure 5e). This may be because the colour of the solution changed from colourless to light yellow and light brown, which affected the colour of the detection strips but did not hinder the determination of H_2_O_2_ consumption. The results showed that the H_2_O_2_ detection strips in an ethanol aqueous solution containing HAuCl_4_ and H_2_PdCl_4_ faded significantly after 12 h compared to the initial solution (as shown in (III) and (IV) of Figure 5e), indicating that H_2_O_2_ played the role of a reducing agent and was consumed during the reduction of HAuCl_4_ and H_2_PdCl_4_. However, there was no significant change in the H_2_O_2_ detection strips measured 12 h before and after the addition of AgNO_3_ and Pd(NO_3_)_2_ to the ethanol aqueous solution system (as shown in (V) and (VI) of Figure 5e), confirming that AgNO_3_ and Pd(NO_3_)_2_ in Figure 5c,d cannot be reduced.

## 4. Conclusions

In this study, we demonstrated a simple, fast, and environmentally friendly method for synthesising noble metal colloids through the interactions between atmospheric-pressure cold plasma and liquids. The experimental results indicated that atmospheric-pressure argon–hydrogen surface DBD plasma can be used to successfully prepare gold, platinum, and palladium colloids within a few minutes. The synthesis process was fast and did not require the use of any other chemical reducing agents. The prepared gold, platinum, and palladium colloids remained stable for 30 d without significant changes. The Au, Pt, and Pd in noble metal colloids exhibited uniform spherical shapes, with average particle sizes of 11.0 ± 2.0 nm, 1.1 ± 0.1 nm, and 3.5 ± 0.3 nm, respectively. In addition, in order to investigate the reduction mechanism of noble metal precursors by surface DBD plasma, we also synthesised noble metal colloids using a plasma-activated ethanol aqueous solution treatment of the noble metal precursors. The comparative results showed that the direct plasma treatment of the ethanol aqueous solutions continuously generated active species such as e^-^_aq_, H, reactive alcohol fragment radicals, and hydrogen peroxide, thereby enabling the rapid reduction of noble metal precursor solutions to obtain gold, silver, platinum, and palladium colloids. The short-lived active species generated by the plasma activation treatment of the ethanol aqueous solution quickly disappeared, and the long-lived species H_2_O_2_ in the activated ethanol aqueous solution oxidised AgNPs and PdNPs, making it impossible to reduce AgNO_3_ and Pd(NO_3_)_2_. The H_2_O_2_ test strip quickly faded after adding HAuCl_4_ and H_2_PdCl_4_ to the plasma-activated ethanol aqueous solution, and the activated ethanol aqueous solution was able to reduce AuCl_4_^−^ and PdCl_4_^2−^ to obtain gold and palladium nanoparticles, indicating that H_2_O_2_ acted as a reducing agent.

## Data Availability

Data are contained within the article and supplementary materials.

## Supplementary Materials

The following supporting information can be downloaded at https://www.mdpi.com/article/10.3390/ma17050987/s1: Figure S1: UV-Vis absorption spectra of HAuCl_4_ precursor solution reduced with different contents of ethanol under the same plasma treatment condition; Figure S2: Time-dependent UV-Vis absorption spectra of the reduced HAuCl_4_ precursor solution at different discharge times with a discharge voltage of 6 kV and 5% PVP; Figure S3: Time-dependent UV-Vis absorption spectra of the reduced HAuCl_4_ precursor solution with different discharge voltages at a discharge time of 7 min and 5% PVP; Figure S4: Time-dependent UV-Vis absorption spectra of HAuCl_4_ precursor solutions reduced with different contents of PVP at a discharge voltage of 6 kV and a discharge time of 7 min; Figure S5: UV-Vis absorption spectra of gold colloid solution without PVP protection over time.
