# Supplementary material for: Rapid Synthesis of Noble Metal Colloids by Plasma–Liquid Interactions
Source: Materials (Basel). 2024 Feb 21;17(5):987. doi: 10.3390/ma17050987 (PMC10933880; doi:10.3390/ma17050987)
Supplement: Supplementary file 1 [file materials-17-00987-s001.zip › materials-2800544-supplementary.pdf]

Supporting Information

Rapid synthesis of noble metal colloids by plasma-liquid interactions

Yuanwen Pang [1], Hong Li [1,*], Yue Hua [1], Xiuling Zhang [1] and Lanbo Di [1,2,3,*]

1 College of Physical Science and Technology, Dalian University, Dalian 116622, China

2 State Key Laboratory of Structural Analysis for Industrial Equipment, Dalian University of Technology, Dalian 116024, China

3 Key Laboratory of Advanced Technology for Aerospace Vehicles of Liaoning Province, Dalian University of Technology, Dalian 116024, China

* Correspondence: lihong10@dlu.edu.cn (H. Li); dilanbo@163.com (L. B. Di).

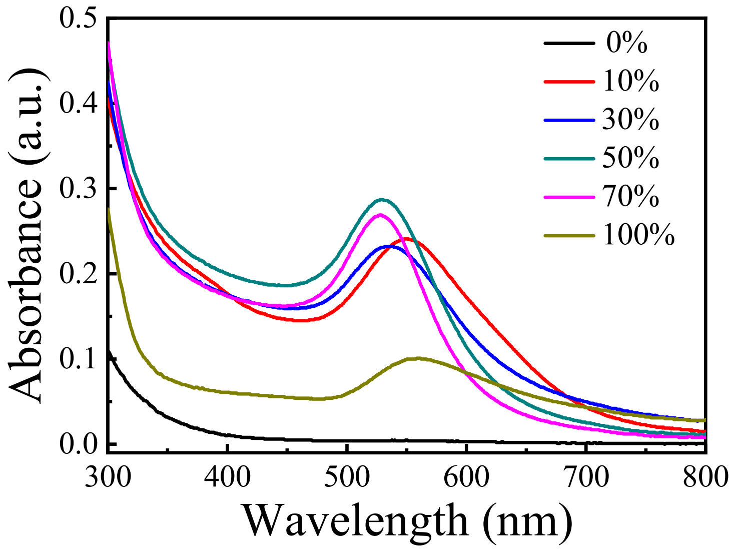

Figure S1. UV-Vis absorption spectra of $HAuCl_4$ precursor solution reduced with different contents of ethanol under the same plasma treatment condition.

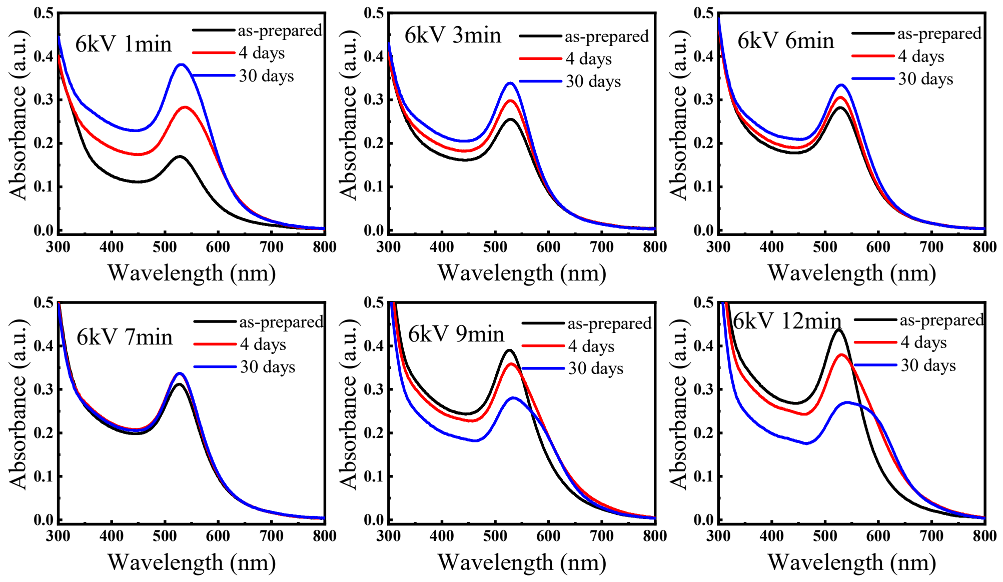

Figure S2. Time-dependent UV-Vis absorption spectra of the reduced $HAuCl_4$ precursor solution at different discharge times with a discharge voltage of 6 kV and 5% PVP.

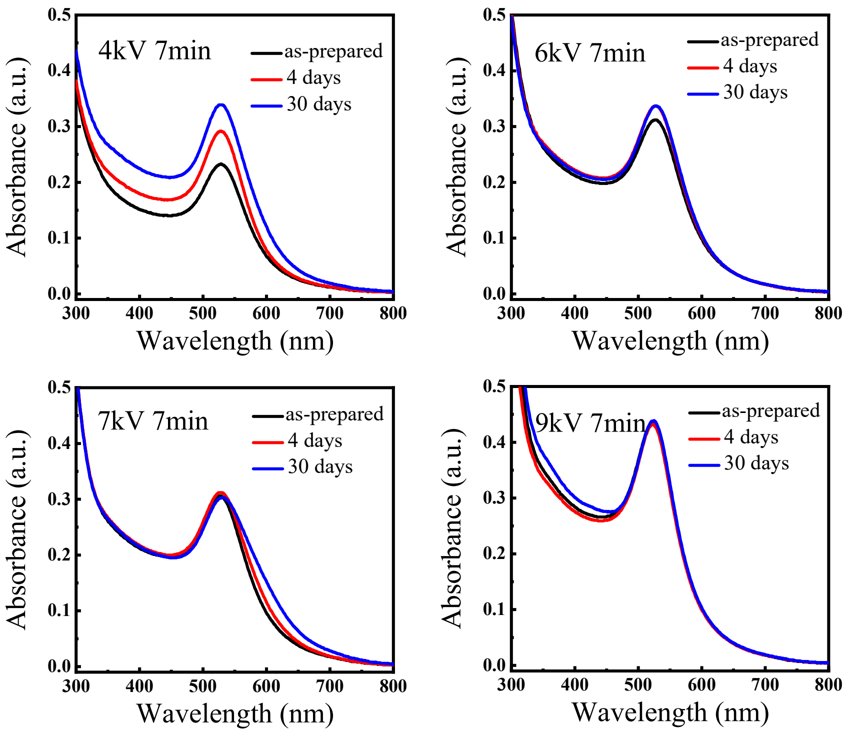

Figure S3. Time-dependent UV-Vis absorption spectra of the reduced $HAuCl_4$ precursor solution with different discharge voltages at a discharge time of 7 min and 5% PVP.

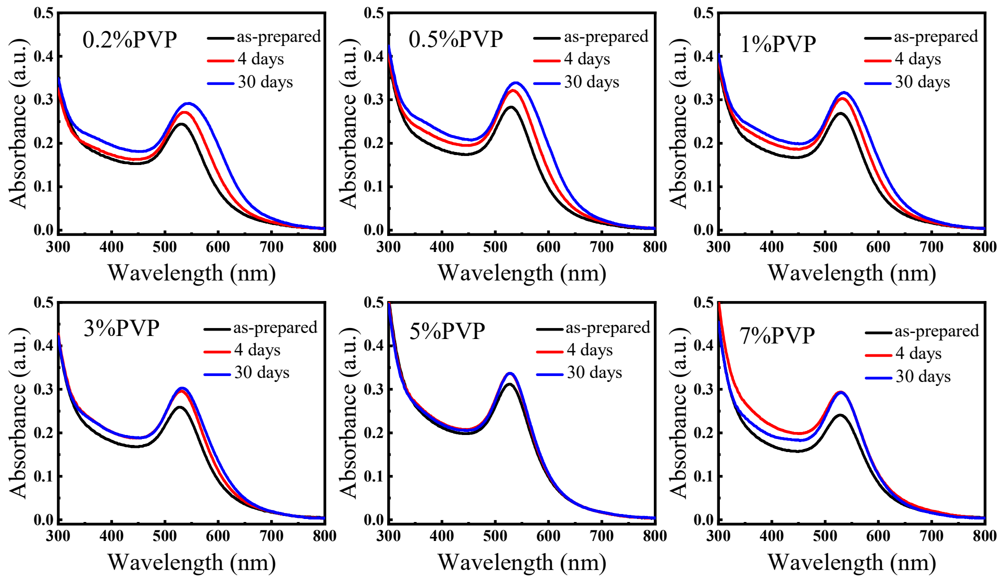

Figure S4. Time-dependent UV-Vis absorption spectra of $HAuCl_4$ precursor solutions reduced with different contents of PVP at a discharge voltage of 6 kV and a discharge time of 7 min.

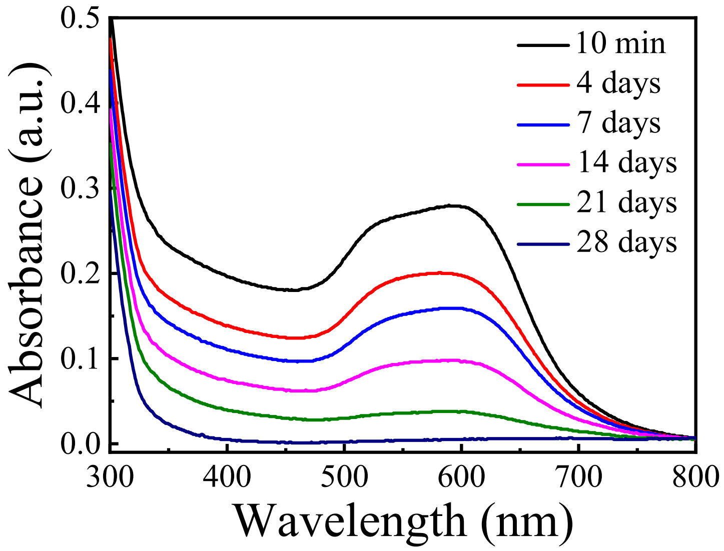

Figure S5. UV-Vis absorption spectra of gold colloid solution without PVP protection over time.
